# Erythroferrone is associated with the hepcidin-to-ferritin ratio and cardiovascular mortality in chronic kidney disease

**DOI:** 10.1093/ckj/sfag075

**Published:** 2026-03-09

**Authors:** Kristin Danielson Pistis, Abdul Rashid Qureshi, Soheir Beshara, Bengt Lindholm, Peter Stenvinkel, Peter Bárány

**Affiliations:** Renal Medicine and Baxter Novum, Department of Clinical Science, Intervention and Technology, Karolinska Institutet, Stockholm, Sweden; Renal Medicine and Baxter Novum, Department of Clinical Science, Intervention and Technology, Karolinska Institutet, Stockholm, Sweden; Department of Molecular Medicine and Surgery, Karolinska Institutet, Stockholm, Sweden; Renal Medicine and Baxter Novum, Department of Clinical Science, Intervention and Technology, Karolinska Institutet, Stockholm, Sweden; Renal Medicine and Baxter Novum, Department of Clinical Science, Intervention and Technology, Karolinska Institutet, Stockholm, Sweden; Renal Medicine and Baxter Novum, Department of Clinical Science, Intervention and Technology, Karolinska Institutet, Stockholm, Sweden

**Keywords:** erythroferrone, erythropoiesis, hepcidin, iron, kidney failure

## Abstract

**Background:**

The role of erythroferrone, a hormone synthesized in erythroid cells that suppresses hepatic production of hepcidin to increase systemic iron availability during erythropoiesis expansion, has not been sufficiently characterized in chronic kidney disease (CKD). Therefore, we evaluated its determinants and its association with mortality risk in patients with CKD stages 3–5.

**Methods:**

In this cross-sectional study with longitudinal follow-up, erythroferrone, hepcidin, ferritin, transferrin saturation (TSAT), C-reactive protein (CRP) and nutritional status were assessed in 377 CKD patients [92 patients with CKD stages 3–4, 210 non-dialysed patients with CKD stage 5 and 75 patients on peritoneal dialysis (PD)]. Regression analyses were performed to investigate the association between erythroferrone and relevant variables. The association between erythroferrone and mortality risk was analysed using Fine–Gray analysis, accounting for kidney transplantation as a competing risk.

**Results:**

Erythroferrone, hepcidin and ferritin were higher in PD patients and in CKD 5 patients as compared with CKD 3–4 patients, whereas the hepcidin-to-ferritin ratio did not differ between the cohorts. In linear regression analysis, erythroferrone was positively associated with the reticulocyte count, CRP and CKD severity and negatively associated with TSAT and the hepcidin-to-ferritin ratio (and hepcidin). Further adjustment for erythropoiesis-stimulating agents (ESAs) revealed that erythroferrone no longer showed an association with the severity of CKD or CRP levels. Finally, erythroferrone was linked to both all-cause mortality and cardiovascular mortality.

**Conclusions:**

Erythroferrone in CKD patients associated with the reticulocyte count and ESA dose and was inversely correlated with the hepcidin-to-ferritin ratio and TSAT, while its relationship with CKD stage may reflect ESA therapy. Higher circulating erythroferrone levels are associated with an increased mortality risk.

## GRAPHICAL ABSTRACT

**GRAPHICAL ABSTRACT:**
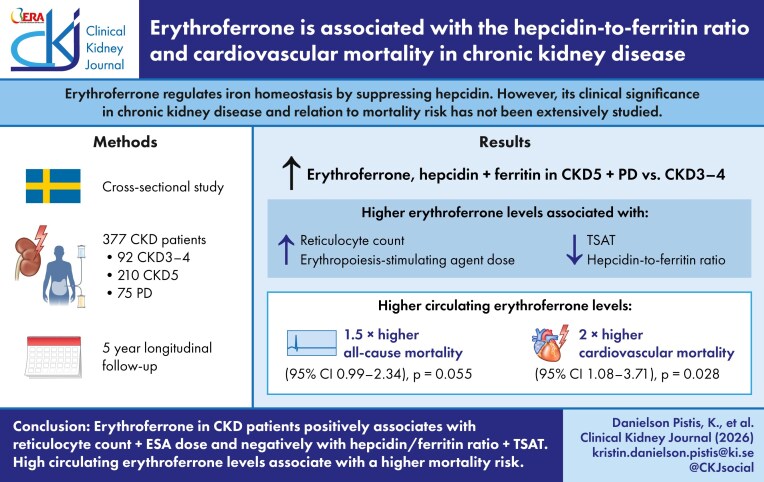

KEY LEARNING POINTS **What was known:**

- Erythroferrone is a hormone synthesized in erythroblasts increasingly expressed in response to erythropoietin that suppresses hepatic expression of hepcidin, a key mediator of iron-restricted anaemia.
- The role of erythroferrone in chronic kidney disease (CKD) in relation to markers of iron homeostasis, erythropoietic activity and physical capacity has hitherto not been elucidated.
- Whether erythroferrone plays a role as a risk marker of poor outcome in the context of CKD has not been extensively studied.

**This study adds:**

- Erythroferrone levels in CKD are inversely associated with iron availability but are unrelated to iron reserves.
- Erythroferrone correlates negatively with the hepcidin-to-ferritin ratio in patients with moderate to severe kidney failure, suggesting that the hepcidin-suppressive effects of erythroferrone are to some extent preserved in CKD.
- Erythroferrone is related to mortality, in particular cardiovascular mortality, in CKD.

**Potential impact:**

- Erythroferrone could potentially hold value when assessing iron homeostasis in patients with CKD.
- Evaluation of erythroferrone levels may potentially contribute to risk stratification in this patient population.

## INTRODUCTION

Dysregulation of iron metabolism is common in chronic kidney disease (CKD) and involves both absolute and functional iron deficiency, both of which may aggravate anaemia and worsen survival [1–3]. Absolute iron deficiency is characterized by depleted iron reserves and is reflected in subnormal ferritin levels. On the other hand, functional iron deficiency entails a situation of insufficient iron availability in which iron-exporting cells are unable to efficiently release iron to the plasma pool, despite normal or large iron reserves, leading to iron-restrictive erythropoiesis.

The latter condition is thought to be induced by hepcidin, a peptide hormone primarily produced in hepatocytes [4]. Hepcidin functions by inhibiting the iron export protein ferroportin, which is located on iron-exporting cells, thereby disrupting efficient recycling of iron [5, 6].

Based on its mechanism of action, and numerous animal and clinical experiments, hepcidin is recognized to play a key role in the pathophysiology of CKD-related anaemia, prompting many studies to attempt to identify its determinants [7, 8]. One such determinant is erythropoietic activity, which through the action of the recently discovered hormone erythroferrone, expressed in high quantity in erythroblasts in response to erythropoietin, inhibits hepatic expression of hepcidin by sequestering bone morphogenetic proteins, central for the regulation of hepatic hepcidin synthesis [9, 10]. By modulating the synthesis of hepcidin, an increase in iron release from iron-exporting cells is enabled so that requirements for erythropoiesis can be met.

Although erythroferrone was shown to play a minor role in sustaining baseline erythropoiesis, it is central during the recovery from anaemia, during which it enhances iron availability to meet the requirements from expanded erythropoiesis [11, 12]. Consistent with its described mode of action, circulating erythroferrone has been shown to increase in parallel with decreases in hepcidin levels upon stimulation with erythropoiesis-stimulating agents (ESA) in patients treated with haemodialysis (HD). Further, erythroferrone (also referred to as myonectin/CTRP15/Fam132B) is also expressed by skeletal muscle cells, and has been attributed a role in regulating various aspects of energy metabolism [13]. Recent experiments indicate that erythroferrone may play a role in muscle wasting, thereby linking it to protein-energy wasting (PEW), commonly observed in advanced CKD [14–16].

The role of erythroferrone remains poorly characterized in the context of CKD [17]. To fill this gap, we assessed determinants of circulating levels of erythroferrone in three cohorts of CKD patients. Finally, we investigated the connection of erythroferrone with all-cause mortality and cardiovascular mortality.

## MATERIALS AND METHODS

This study examined baseline erythroferrone in 377 CKD stage 3–5 patients from three longitudinal cohort studies carried out at Karolinska University hospital. Participants (aged 19–87) were clinically stable and followed for up to 60 months or until death or kidney transplantation. Exclusion criteria comprised patients under 18 years of age, clinical signs of infection, or those unwilling to participate. All provided written consent, and the protocols were approved by the regional ethical review board in Stockholm and in accordance with the Declaration of Helsinki. The study cohorts’ characteristics have been previously outlined [18–20]. A complete description of the methods can be found in the Supplementary material.

### Statistical analyses

Variables were expressed as either median (interquartile range (IQR)) or mean ± SD. The comparison between groups was executed using non-parametric tests, specifically the Mann-Whitney U test for continuous variables, Chi-square test for nominal variables, and the Kruskal-Wallis test for multiple groups. To determine associations between variables, Spearman’s rank correlation was utilized. For assessing independent predictors of erythroferrone levels, multiple linear regression models were applied, with non-normally distributed variables being log-transformed before inclusion in the model.

To analyse competing risk, a Fine-Gray analysis was conducted to calculate cumulative incidence curves for survival, considering kidney transplantation as a competing risk. Patients were divided into tertiles based on erythroferrone levels, and mortality risk estimates for the high tertile were compared with the remaining patients. These were expressed as sub-distribution hazard ratios (s-HR) with 95% confidence intervals (95% CI). Lastly, a modified Poisson regression was used to calculate the cubic spline curve for erythroferrone and its association with mortality.

Statistical analyses were carried out using Stata Now 19.5 (Stata Corporation, College Station, TX, USA), SAS version 9.4 update 8 (SAS Campus Drive, Cary, NC, USA), and SPSS version 27. A *P*-value of less than 0.05 was considered statistically significant.

## RESULTS

Baseline measurements of erythroferrone were available for 377 patients, of which 92 patients had CKD stage 3–4, 210 patients had CKD stage 5 starting on dialysis (CKD5-ND), and 75 CKD 5 patients were treated with PD. Patient characteristics are summarized in Table 1.

**Table 1:** Clinical characteristics of 377 patients with clinically stable CKD.

Characteristics	Total (*N* = 377)	CKD 3–4 (*n* = 92)	CKD 5 (*n* = 210)	PD (*n* = 75)	*P*-value
Age (years)	59 (48–68)	60 (47–70)	57 (47–65)	65 (56–77)	<.05
Males, *n* (%)	259 (69)	66 (72)	142 (68)	51 (68)	.79
Diabetes mellitus, *n* (%)	112 (30)	28 (30)	64 (31)	20 (27)	.77
Cardiovascular disease, *n* (%)^a^	127 (34)	22 (24)	83 (40)	22 (29)	<.05
Framingham Risk Score	22.1 (9.7–35.6)	25.6 (10.2–49.2)	16.8 (8.1–30.0)	26.5 (16.4–43.3)	<.01
GFR (ml/min/1.73 m^2^)^b^	6 (4–11)	25 (15–32)	6 (5–8)	3 (1–4)	<.05
Markers of nutritional status					
PEW (SGA >1), *n* (%)^c^	94 (26)	2 (2)	55 (29)	37 (53)	<.05
Body mass index (kg/m^2^)	25 ± 4	27 ± 5	25 ± 4	25 ± 4	<.05
Handgrip strength (%), %^c^	84 (67–100)	97 (80–110)	82 (65–98)	73 (59–88)	<.01
Treatment characteristics					
ESA therapy, *n (%)*	265 (70)	12 (13)	186 (89)	67 (89)	<.05
Weekly ESA dose/kg (IU/w/kg)	75 (49–115)	56 (33–68)	77 (50–116)	72 (46–123)	.050
Iron supplementation, *n* (%)	154 (41)	16 (17)	113 (54)	25 (33)	<.05
RAS inhibitor, *n* (%)	264 (71)	72 (78)	149 (72)	43 (57)	<.05
β-blocker, *n* (%)	244 (66)	49 (53)	146 (72)	49 (65)	<.05
Calcium blocker, *n* (%)	184 (50)	42 (46)	119 (60)	23 (31)	<.05

Numbers shown are mean, ± SD, median (interquartile range), or absolute numbers (%). GFR: glomerular filtration rate; PEW: protein energy wasting; SGA: subjective global assessment; ESA: erythropoiesis-stimulating agents; RAS: renin–angiotensin system inhibitors.

a According to clinical history or presence of cardiovascular disease at inclusion.

b Estimated based on the Chronic Kidney Disease Epidemiology Collaboration equation for patients without dialysis and on the mean of renal creatinine and urea clearance from 24-hour urine collection in patients on peritoneal dialysis.

c Available in 355 patients.

### Baseline levels of circulating erythroferrone levels

**Figure 1:**
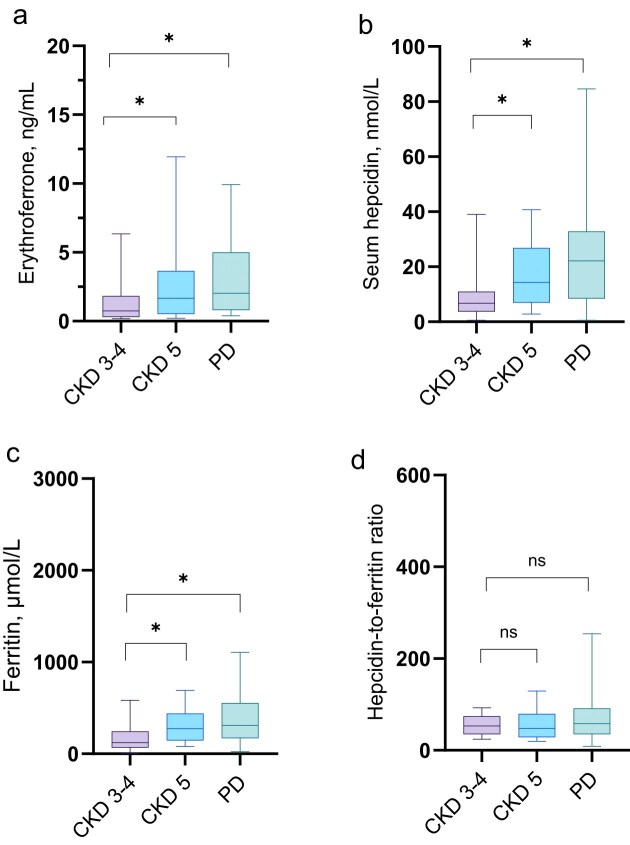
Circulating levels of (a) erythroferrone, (b) hepcidin and (c) ferritin and (d) the hepcidin-to-ferritin ratio according to CKD cohort. The box and whisker plots represent the 10th and 90th percentiles of the data. *Denotes *p* < .05 (*p*-values adjusted by the Bonferroni correction).

Erythroferrone increased with declining kidney function. Median (IQR) erythroferrone was more than twofold higher in patients with CKD5-ND 1.7 (0.5–3.6) ng/mL and PD 2.0 (0.8–5.0) ng/mL, respectively, than in patients with CKD stage 3–4, 0.7 (0.3–1.8) ng/mL; *P* < 0.05 for both comparisons (Fig. 1).

Hepcidin increased in a similar fashion. Median (IQR) was more than twofold higher in patients with CKD5-ND, 14.3 (7.0–26.7) nmol/L and PD, 22.1 (8.5–32.8) nmol/L, respectively, as compared to patients with CKD stage 3–4, 6.7 (3.7–11.0) nmol/L; *P* < 0.05 for both comparisons.

In contrast, the hepcidin-to-ferritin ratio was similar in the three cohorts: In CKD5-ND, 49 (31–80), PD, 58 (35–92), and CKD stage 3–4, 53 (35–74).

In line with a higher degree of iron loading in patients with more severe kidney failure, ferritin was more than twofold higher in patients with CKD5-ND, 274 (146–439) µg/L and PD, 300 (170–539) µg/L, respectively, as compared to patients with CKD stage 3–4, 121 (70–243) µg/L; *P* < 0.05 for both comparisons.

### Univariate correlations between erythroferrone and laboratory parameters

The results from univariate correlations assessed with Spearman’s *rho* are depicted in Table S3. Erythroferrone correlated positively with age (rho = 0.20; *P* < 0.01), the reticulocyte count (rho = 0.20; *P* < 0.01), weekly ESA dose (rho = 0.31; *P* < 0.01), C-reactive protein (CRP; rho = 0.24 (*P* < 0.01), interleukin-6 (IL-6; rho = 0.23; *P* < 0.01), and negatively with the transferrin saturation (TSAT; rho= –0.24; *P* < 0.01), hepcidin (rho= –0.17; *P* < 0.01), the hepcidin-to-ferritin ratio (rho= –0.32; *P* < 0.01), P-iron (rho= -0.27; *P* < 0.01), HGS% (rho= –0.20; *P* < 0.01), albumin (rho= –0.17; *P* < 0.01), and GFR (defined as above or below 15 mL/min/1.73m^2^; rho= –0.17; *P* = 0.001). The negative association between erythroferrone and hepcidin was however not present in the subgroup of patients without ESA-treatment (n = 112; rho = –0.12, *P* = 0.21), whereas the association between erythroferrone and the hepcidin-to-ferritin ratio remained in this subgroup (rho = –0.27, *P* < 0.05).

### Correlations between erythroferrone and categorical factors

Assessment of erythroferrone based on categorical factors revealed that the presence of PD, PEW (SGA > 1), cardiovascular disease, and treatment with iron, and ESA, respectively, associated with elevated levels of erythroferrone (Table S2).

### Determinants of serum erythroferrone

In a multiple linear regression model we incorporated parameters that were associated with erythroferrone in unadjusted analyses (see Table S2 and Table S3). These analyses showed the hepcidin-to-ferritin ratio and treatment with PD to be the factors most strongly related to erythroferrone with adjusted regression coefficients of –0.24 (*P* < 0.001) and 0.24 (*P* < 0.001), respectively (Table 3). Hepcidin also associated with erythroferrone (adjusted β = –0.23; *P* < 0.001) when incorporated into the model instead of the hepcidin-to-ferritin ratio. Iron availability (TSAT), was negatively correlated with erythroferrone (β= –0.17; *P* = 0.003) whereas CRP was positively correlated with erythroferrone (β = 0.14; *P* = 0.013). The reticulocyte count showed a weaker association with erythroferrone levels (β = 0.11; *P* = 0.044). Of note, when the weekly ESA dose was included into the model instead of the reticulocyte count, the associations of PD and CRP, respectively, with erythroferrone were lost. Neither PEW_SGA_ nor HGS% was significantly correlated with erythroferrone.

In a subgroup of patients (n = 166) with available fT3/fT4 ratio measurements, we observed a negative association between this marker and erythroferrone levels (β = –0.20; *P* = 0.025; R² = 0.23). After adjusting for the fT3/fT4 ratio, the significant associations of iron availability (TSAT) and CRP with erythroferrone were no longer observed (Table S4).

### Serum erythroferrone and mortality

**Figure 2:**
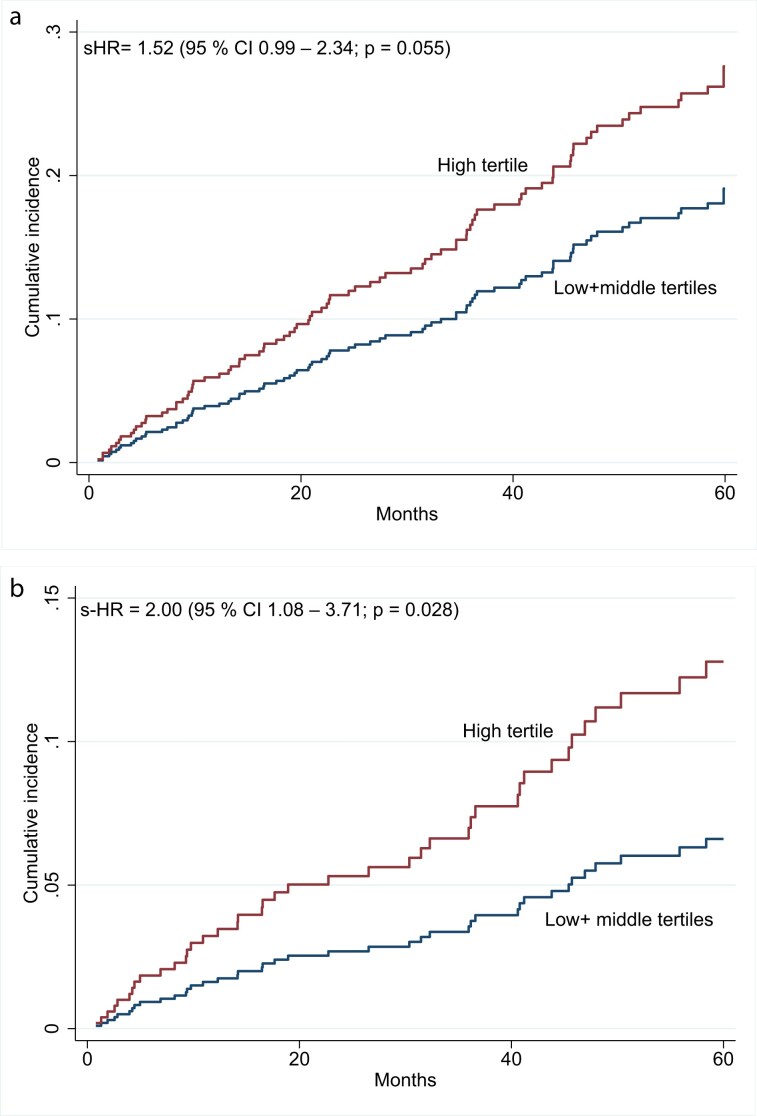
Cumulative incidence of (a) all-cause mortality and (b) cardiovascular mortality among CKD patients within the high tertile of circulating erythroferrone versus patients within low and middle tertiles. Mortality risk was assessed with Fine–Gray competing risk analysis and expressed as the subdistribution hazard ratio (s-HR) and 95% confidence interval, following adjustments for CKD cohort, Framingham Risk Score, protein-energy wasting (as defined by subjective global assessment), transferrin saturation and C-reactive protein.

During a follow-up of up to 60 months, with no loss of follow-up, 104 (28%) patients died (11 in the CKD 3–4 cohort, 59 in the CKD5-ND cohort, and 34 in the PD cohort) and 136 (36%) were transplanted (one in the CKD 3–4 cohort, 115 in the CKD5-ND cohort, and 20 in the PD cohort). All-cause and cardiovascular mortality, assessed with Fine-Gray analysis applying kidney transplantation as competing risk, was assessed by comparing patients of the highest tertile of erythroferrone (> 2.49 ng/mL) with the remaining patients (low and middle tertile combined). The cumulative incidence of all-cause mortality was found to be significantly higher in the high tertile of erythroferrone as compared to the remaining patients, with adjustments for CKD cohort, Framingham risk score, PEW_SGA_, and TSAT (s-HR = 1.55 (1.01–2.38); *P* = 0.045). After further adjustment for inflammatory activity (CRP), this association was no longer statistically significant (s-HR = 1.52 (0.99–2.34); *P* = 0.055) (Fig. 2).

Additionally, the high tertile of erythroferrone was associated with a higher cumulative incidence of cardiovascular mortality as compared to the remaining patients, even after adjustments for CKD-cohort, Framingham risk score, PEW_SGA_, TSAT, and CRP (s-HR = 2.00 (1.08–3.71); *P* = 0.028) (Fig. 2)_._ The result of each analysis is shown in Supplemental material (Table S5a and b).

Of note, high levels of erythroferrone levels remained significantly associated with mortality (all-cause mortality and cardiovascular mortality, respectively) when the CKD 3–4 cohort was excluded from the analyses (data not shown).

## DISCUSSION

This cross-sectional study with longitudinal follow-up shows that in patients with moderate to advanced CKD, increased circulating levels of erythroferrone were positively and independently associated with the reticulocyte count and ESA dose and inversely associated with the hepcidin-to-ferritin ratio and systemic iron availability as assessed by TSAT. Higher levels of erythroferrone were significantly associated with increased risk of mortality, in particular cardiovascular mortality.

Erythroferrone is produced by erythroblasts upon stimulation with erythropoietin [11]. In situations of expanded erythropoiesis, erythroferrone contributes by increasing iron availability so that erythropoietic iron requirements can be met [12]. Since hepcidin plays a central role in the pathogenesis of renal anaemia, it has been hypothesized that increasing erythroferrone levels could counteract excess hepcidin in CKD [21–23].

We observed that erythroferrone levels increased as CKD progressed (Fig. 1). However, upon adjusting for inflammatory activity, the hepcidin-to-ferritin ratio, iron availability, and weekly ESA-dosage, the association with CKD severity was no longer significant (Table 3). This suggests that low GFR alone does not directly correlate with elevated erythroferrone levels in late-stage CKD. Instead, it appears to be influenced by other factors, including variations in the intensity of ESA treatment. This observation is consistent with previous studies in mice, healthy individuals, and CKD patients, all showing that ESA stimulates the expression of erythroferrone [24, 25].

The relation between erythroferrone and erythropoietic activity was confirmed by its association with the reticulocyte count. Whether an early measurement of erythroferrone post ESA treatment may add value in predicting whether the ESA treatment has its intended effect in raising haemoglobin levels in CKD-patients remains to be evaluated [26, 27].

Hepcidin is considered a key player in the pathogenesis of renal anaemia by promoting iron-restrictive erythropoiesis [28]. It has been hypothesized that due to bone marrow erythropoiesis typically being hypoproliferative in renal failure, characterized by a low output of erythroblasts, insufficient amounts of erythroferrone contribute to hepcidin excess in the uremic milieu [21].

In the present study, we found an inverse association between erythroferrone and hepcidin that withstood multivariate adjustments. However, in the subgroup of patients devoid of ESA (n = 113), the univariate association between erythroferrone and hepcidin association was not present, a finding consistent with a previous study in CKD-patients without ESA-treatment [24]. Consequently, the association between erythroferrone and hepcidin may stem from the expansion of erythropoiesis stimulated by ESA.

However, in order to appropriately interpret hepcidin levels they need to be considered in relation to ferritin levels, the major determinant of hepcidin levels in clinically stable CKD patients [7, 8, 29, 30]. Therefore, we also considered the hepcidin-to-ferritin ratio, which reflects the adequacy of hepcidin expression in relation to ferritin levels, determined by the size of iron stores and to some extent by chronic inflammation, and found its negative association to erythroferrone to be present in both the whole cohort, and in the subgroup of patients without ESA treatment. The association remained present in each of the various cohorts (data not shown). It therefore seems that the counteractive effects of erythroferrone on hepcidin levels, at least to some extent, remain intact in patients with moderate to severe CKD.

Although hepcidin and ferritin rose as CKD severity increased, in keeping with previous reports, the hepcidin-to-ferritin ratio did not differ between the cohorts suggesting that hepcidin was not excessively expressed in the later CKD stages as compared to CKD 3–4 when normalized to the ferritin levels, possibly attributed to the hampering effects of erythroferrone on hepcidin expression [7]. It therefore seems that ferritin levels constitute a key driver of the hepcidin increase in kidney failure.

Closely related to erythropoietic activity is systemic iron availability (reflected by TSAT), which was shown to be negatively associated with erythroferrone, even after multivariate adjustments. This finding also held true when patients on ESA were excluded from the regression model (data not shown) and was therefore not merely an effect mediated by ESA.

Of note, erythroferrone levels were insensitive to the size of iron reserves, as measured by ferritin. Erythroferrone therefore seems to be associated with systemic iron availability rather than with iron reserves, but also with erythropoietic activity. In situations of limited iron availability, the sensitivity of erythroid cells to erythropoietin is reduced through inactivation of transferrin receptor 2, such that continuous maturation of erythroid progenitor cells is stagnated [31, 32]. Therefore, the increase in erythroferrone in systemic iron deficiency may not be predominantly attributed to an elevated output of erythroblasts but perhaps more importantly due to an increase in the expression of erythroferrone per erythroblast cell [12].

Although we found a significant correlation between erythroferrone and inflammatory activity (CRP) which withstood adjustments for CKD-severity, the reticulocyte count, the hepcidin-to-ferritin ratio (and hepcidin), and iron availability, it was lost when additionally adjusting for the weekly ESA dose suggesting that ESA therapy mediates this association [17]. However, an association between erythroferrone and CRP was also present in patients devoid of ESA (Spearman’s *rho*: 0.24; *P* = 0.03, n = 112), and a correlation may therefore exist independently of ESA therapy.

Erythroferrone was previously shown to be increasingly expressed in skeletal muscle tissue from cachectic patients, and we therefore investigated the relation between erythroferrone and markers reported to reflect nutritional status and physical capacity, two entities closely intertwined [16, 33, 34]. These included SGA_PEW_, HGS%, and the fT3/fT4 ratio, where the latter is a marker previously shown to associate with physical performance [35]. In a subgroup of patients with these measurements available, we found the fT3/fT4 ratio to negatively associate with erythroferrone, potentially reflective of a situation in which increased erythroferrone expression coincides with decelerated T4 to T3 conversion and impaired T3 signalling in peripheral tissues.

Interestingly, when adjustments were made for the fT3/fT4 ratio, the negative association between erythroferrone and TSAT was modified. It may therefore be speculated that the mechanism linking low fT3/fT4 ratio to high erythroferrone levels lies in the same pathway that links low iron availability to an increase in erythroferrone expression. Such a pathway could involve iron deficiency-induced stabilization of HIF1 with ensuing increased erythropoietin signalling and a resulting increase in erythroferrone output [36, 37]. Further, stabilization of HIF1 induces type 3 deiodinase-mediated inactivation of T3, which would lower the fT3/fT4 ratio [38].

Finally, higher levels of erythroferrone were associated with all-cause mortality although this association was no longer statistically significant when additional adjustments for inflammatory activity were performed, suggesting that high levels of erythroferrone and inflammatory activity are linked to all-cause mortality via a common pathway [39]. However, for cardiovascular mortality, the association with high levels of erythroferrone remained significant even after adjustments for inflammatory activity (CRP). Of note, additional adjustment for the weekly ESA dose in those patients treated with ESA weakened this association, bringing into question the role of ESA in mediating the relation between erythroferrone and CVD mortality.

Consistent with the findings of the present study, a previous study comprising patients treated with haemodialysis (HD) and non-dialysis patients found an association between high erythroferrone levels and the combined endpoint of all-cause mortality and cardiovascular events [17].

The mechanism linking high levels of erythroferrone to cardiovascular mortality cannot be elucidated based on the present study. However, we found erythroferrone associated with circulating levels of NT-pro BNP (Spearman’s ρ = 0.27; *P* = 0.02; *n* = 75), a biomarker that holds diagnostic value in the management of heart failure [40–42]. More studies are required to clarify the role of erythroferrone in the context of cardiovascular disease and CKD.

There are some limitations to this study. First, given its cross-sectional design, conclusions on causality cannot be drawn. Second, patient cohorts differed as regards CKD stage and treatment. Nevertheless, this study comprised a relatively large number of study subjects across various stages of CKD, which allowed for multivariate adjustments in an attempt to identify predictors of erythroferrone, a hormone that has been suggested to hold promise for the treatment of renal anaemia but as yet is poorly characterized in the CKD population [21–23]. An additional strength of the present study is that the analysis of the hepcidin concentration was performed with mass spectrometry, which allows for the quantification of hepcidin-25 exclusively without measuring other hepcidin isomers that may increase disproportionally in CKD [28].

In conclusion, this study involving 377 CKD patients found that erythroferrone levels were inversely related to iron availability and the hepcidin-to-ferritin ratio and positively correlated with the dose of ESA therapy. Additionally, erythroferrone was linked to markers of malnutrition and muscle strength; however, only the relationship with the FT3/FT4 ratio remained significant after multivariate adjustments. Lastly, we report that higher circulating levels of erythroferrone were associated with increased mortality risk.

**Table 2:** Laboratory characteristics of 377 patients with clinically stable CKD.

Characteristics	Total (*N* = 377)	CKD 3–4 (*n* = 92)	CKD 5 (*n* = 210)	PD (*n* = 75)	*P*-value
Haemoglobin (g/L)	113 (102–125)	115 (104–127)	113 (103–125)	110 (101–121)	<.05
Reticulocyte count (10^9^/L)^a^	53 (39–67)	59 (46–74)	51(36–66)	48 (36–60)	<.05
Serum albumin (g/L)	34 (31–37)	37 (36–39)	34 (31–37)	32 (28–35)	<.05
iPTH (ng/L)	182 (98–327)	105 (74–145)	229 (120–360)	264 (144 -429)	<.05
CRP (mg/L)	3.4 (1.2–9.0)	2.8 (1.1–5.4)	4.1 (1.3–10.0)	4.0 (1.3–9.0)	<.05
IL-6 (pg/mL)^b^	5.1 (2.9–9.1)	2.7 (2.3–3.5)	6.5 (3.4–9.8)	6.3 (3.9–9.6)	<.05
Erythroferrone (ng/mL)	1.5 (0.5–3.3)	0.7 (0.3–1.8)	1.7 (0.5–3.6)	2.0 (0.8–5.0)	<.05
Hepcidin (nmol/L)^c^	11.5 (5.8–25.2)	6.7 (3.7–10.9)	14.3 (7.0–26.7)	22.1 (8.5 -32.8)	<.05
Hepcidin-to-ferritin ratio^d^	52 (32–82)	53 (35–74)	49 (31–80)	58 (35–91)	.19
Ferritin (μg/L)	241 (116–410)	121 (70–241)	274 (147–439)	300 (170–539)	<.05
TSAT (%)^e^	23 (17–30)	26 (21–31)	20 (15–28)	27 (18–37)	<.05
Iron (μmol/L)^f^	12 (8–15)	14 (12–17)	10 (7–13)	13 (9–18)	<.05
Cholesterol (mmol/L)	4.6 (3.9–5.5)	5.2 (4.5–5.9)	4.3 (3.6–5.2)	4.9 (4.3–5.8)	<.05
FT3 (pmol/L)^g^	3.3 (2.4–4.1)	NA	2.5 (1.9–3.4)	3.8 (3.5–4.4)	<.05
FT4 (pmol/L)^g^	12 (1.1–15.0)	NA	1.2 (1.0–15.0)	13.0 (11.0–15.0)	<.05
FT3/FT4 ratio^g^	0.3 (0.3–1.6)	NA	1.4 (0.3–2.0)	0.3 (0.2–0.3)	<.05
*N*-terminal-proBNP (ng/L)^h^	3030 (1150–6970)	NA	NA	3030 (1150–6970)	–

Values are presented as median (IQR).

iPTH: intact parathyroid hormone; FT3: free triiodothyronine; FT4: free thyroxine.

a Available in 354 patients.

b Available in 330 patients.

c Reference values for serum levels of hepcidin-25 (nmol/l; median values, 95% reference range) measured with mass spectrometry: men 4.7 (<0.5–15.5), women 3.8 (<0.5–15.4). Reference: www.hepcidinanalysis.com.

d Reference values for the hepcidin-to-ferritin ratio (median, 95% reference range) measured with mass spectrometry: men 28.2 (3.1–92.7), pre-menopausal women 37.6 (3.2–176.4), post-menopausal women 42.7 (9.6–150.9). Reference: www.hepcidinanalysis.com.

e Available in 342 patients.

f Available in 362 patients.

g Available in 166 patients.

h Available in 75 patients.

**Table 3:** Multiple linear regression with log-normalized serum erythroferrone as a dependent variable^a^.

Variable	β	Lower limit	Upper limit	*P*-value	VIF
log age	0.10	−0.06	1.12	.081	1.2
gender	−0.02	−0.17	1.11	.686	1.0
CKD 5 versus CKD 3–4	0.15	0.03	0.36	.023	1.7
PD versus CKD 3–4	0.24	0.18	0.57	<.001	1.7
log RET-C	0.11	0.01	0.77	.044	1.3
log hepcidin-to-ferritin ratio	−0.24	−0.76	−0.29	<.001	1.3
log TSAT	−0.17	−0.89	−0.18	.003	1.2
log CRP	0.14	0.04	0.29	.013	1.3
log HGS%	−0.02	−0.65	0.45	.716	1.3

Adjusted *R*^2^ = 0.20; *n* = 319 CKD patients.

a Regression analyses with log-normalized serum erythroferrone as a dependent variable.

Exchange of log-normalized HGS% for SGA_PEW_ did not significantly change the outcome of the model [β=0.03 (CI −0.12–0.20), *P* = 0.64; *R*^2^ = .21; VIF = 1.3].

Substitution of the log-normalized hepcidin-to-ferritin ratio for the log-normalized serum hepcidin-25 [standardized β = −0.23 (CI −0.48 to −0.16), *P* = <.001; VIF = 1.3] did not significantly change the model (*R*^2^ = 0.19).

Substitution of the log-normalized RET-C for the log-normalized values of weekly ESA dose showed the weekly ESA dose to associated with the log-normalized values of serum erythroferrone [β = 0.21 (CI 0.19–0.79), *P* = .002; *R*^2^ = 0.20; VIF = 1.2]. This implied a loss of association between the log-normalized serum erythroferrone and the log-normalized CRP [β = 0.12 (CI −0.01–0.29), *P* = .068], CKD 5 [β = 0.07 (CI −0.25–0.45), *P* = .58] and PD [β = 0.20 (CI −0.09–0.65), *P* = .14].

β: standardized coefficient **B**eta; PD: peritoneal dialysis; RET-C: reticulocyte count; CRP: high-sensitivity C-reactive protein, TSAT: transferrin saturation; HGS%: hand-grip strength %; SGA: subjective global assessment; ESA: erythropoiesis-stimulating agent; VIF: Variance Inflation Factor.

## Supplementary Material

sfag075_Supplemental_Files

## Data Availability

The data underlying this article will be shared upon reasonable request to the corresponding author.
