# Supplementary material for: Erythroferrone is associated with the hepcidin-to-ferritin ratio and cardiovascular mortality in chronic kidney disease
Source: Clin Kidney J. 2026 Mar 9;19(5):sfag075. doi: 10.1093/ckj/sfag075 (PMC13136888; doi:10.1093/ckj/sfag075)
Supplement: sfag075_Supplemental_Files [file sfag075_supplemental_files.zip › Supplementary material 3rd Dec 2025_CLEAN VERSION.docx]

**Supplementary material**

**Complete Methods**

*Study subjects*

*CKD Stage 3–4 (n=92):* The study participants were recruited from 2001 to 2011. At baseline, the average age of the participants was 58 years, 72% were men, 30% had diabetes mellitus, and 24% had cardiovascular disease. The median estimated glomerular filtration rate was 25 ml/min/1.73^2^ (ranging from 15 to 32 ml/min/1.73^2^). The causes of CKD were glomerulonephritis (23%), diabetes mellitus (11%), hypertension/renal vascular disease (2 %), and other or unknown causes (64 %). 13 % received ESA therapy and 17 % were supplemented with iron[1].

*CKD Stage 5–Non-Dialysis (n=210):* Patients with CKD stage 5 were enrolled between 2007 and 2008 as part of an ongoing study of incident dialysis patients who were about to start maintenance dialysis. The mean age was 55 years, 68% were men, 31 % had diabetes mellitus, and 40% had cardiovascular disease. ESA and iron supplementation were prescribed to 89 % and 54 % of the study participants, respectively[2].

*CKD Stage 5–Dialysis (n=75):* Data from patients undergoing peritoneal dialysis, recruited between 2008 and 2011, were collected from a study aiming to evaluate markers of inflammation in patients undergoing peritoneal dialysis. Median K_T_/V_urea_ was 2.2 (interquartile range, 1.8-2.5) and median dialysis vintage (time on dialysis) was 11.3 (6.0 – 28.8) months. The residual glomerular filtration rate, quantified from the renal creatinine and urea clearances by means of 24-hour urine collection, was 2.8 (1.4 –4.6) mL/min/1.73 m^2^. The mean age was 64, 68% were men, 27 % had diabetes mellitus, and 29 % had cardiovascular disease. Causes of kidney failure were glomerulonephritis (15 %), diabetes mellitus (13 %), hypertension/ renal vascular disease (16 %), and other or unknown causes (56 %). 89 % received ESA therapy to maintain the hemoglobin level between 100 and 120 g/L. 33 % received iron supplementation[3].

*Laboratory Protocol*

Blood samples were collected after an overnight fast, and plasma and serum were separated and stored at -70°C unless analyzed immediately. Biochemical analyses were performed using routine methods at Karolinska University Hospital. Hepcidin was measured in stored serum using mass spectrometry whereas erythroferrone was measured using a validated ELISA method (Intrinsic Life Sciences, La Jolla, CA, USA), also in stored serum[4]. Hepcidin was adjusted to the ferritin level, yielding the hepcidin-to-ferritin ratio, a marker reflecting hepcidin expression adequacy in relation to iron reserves. Complete details of the laboratory protocol are found in the Supplementary material.

*Data collection*

Demographic information, cause of CKD, residual kidney function (GFR; calculated by the Chronic Kidney Disease Epidemiology Collaboration (CKD-EPI) equation in non-dialyzed patients, and from the mean of renal creatinine and urea clearances of 24-hour urine collection in patients with peritoneal dialysis (PD)), presence of kidney replacement therapy, comorbidities, anthropometric data, nutritional status, and concomitant medical treatment, were recorded. Additionally, we calculated the weekly ESA dose per kilogram body weight (IU/week/kg) for patients receiving ESA treatment. A factor of 200 was applied to convert darbepoetin to the corresponding epoetin dose. Survival time was calculated from the day of examination until either the occurrence of death or censoring for renal transplantation. Cardiovascular mortality was defined as death caused by myocardial ischemia, myocardial infarction, heart failure, sudden cardiac arrest, or death due to a cerebrovascular accident. Missing causes of death were reported as missing.

*Anthropometric evaluation*

Handgrip strength (HGS) was quantified with a Harpenden® Handgrip Dynamometer (Yamar, Jackson, MI, USA). Absolute HGS values were normalized to the average HGS value in controls rendering the HGS% value.

Nutritional status was evaluated using the 4-point subjective global assessment (SGA) scale. Patients were categorized as follows: (1) normal nutritional status, (2) mild PEW, (3) moderate PEW, or (4) severe PEW. PEW was defined as a SGA score greater than 1.

The fT3/fT4 ratio, a marker shown to be associated with muscle mass and physical capacity, was included for assessment of its relation to erythroferrone [5-7].

| Table S1. Baseline characteristics of 377 CKD patients according to tertiles of erythroferrone | | | | | | | | |  |  |  |
| --- | --- | --- | --- | --- | --- | --- | --- | --- | --- | --- | --- |
|  |  |  | | |  |  |  | | | |  |
|  | **All patients** | | **Low**  **tertile** | **Middle**  **tertile** | | **High**  **tertile** | | **p-value** |  |  |  |
|  |  | |  | |  |  | |  |  |  |  |
| Variables | n=377 | | n=124 | | n=125 | n=128 | |  |  |  |  |
|  |  | |  | |  |  | |  |  |  |  |
| Age, years | 59 (48-68) | | 57 (42-67) | | 56 (47-67) | 63 (54-70) | | <0.001 |  |  |  |
|  |  | |  | |  |  | |  |  |  |  |
| Males, n (%) | 259 (69) | | 84 (68) | | 88 (71) | 87 (68) | | 0.83 |  |  |  |
|  |  | |  | |  |  | |  |  |  |  |
| Diabetes mellitus, n (%) | 112 (30) | | 32 (26) | | 35 (28) | 45 (35) | | 0.25 |  |  |  |
|  |  | |  | |  |  | |  |  |  |  |
| Cardiovascular disease, n (%)^a^ | 127 (34) | | 30 (25) | | 43 (38) | 54 (43) | | 0.011 |  |  |  |
|  |  | |  | |  |  | |  |  |  |  |
| PEW_SGA,_ n (%) | 94 (26) | | 22 (19) | | 26 (23) | 46 (37) | | 0.005 |  |  |  |
|  |  | |  | |  |  |  | | | |  |
| Handgrip strength% (%) | 84 (67-100) | | 88 (70-102) | | 85 (72-100) | 74 (61-93) | | 0.001 |  |  |  |
| Hemoglobin (g/L) | 113 (102-125) | | 115 (104-127) | | 113 (103-125) | 110 (101-121) | | 0.11 |  |  |  |
|  |  | |  | |  |  | |  |  |  |  |
| Reticulocyte count (10^9^/L) | 53 (39-67) | | 49 (38-60) | | 52(36-66) | 58 (41-74) | | 0.007 |  |  |  |
|  |  | |  | |  |  | |  |  |  |  |
| Glomerular filtration rate^b^ (mL/min/1,73m^2^) | 6 (4-11) | | 8 (5-17) | | 6 (4-10) | 6 (4-9) | | <0.001 |  |  |  |
|  |  | |  | |  |  | |  |  |  |  |
| C-reactive protein (mg/L) | 3.4 (1.2-9.0) | | 2.3 (1.0-5.3) | | 3.9 (1.5-9.0) | 5.6 (1.3-12.8) | | <0.001 |  |  |  |
|  |  | |  | |  |  | |  |  |  |  |
| Erythroferrone (ng/mL) | 1.5 (0.5-3.3) | | 0.3 (0.2-0.5) | | 1.4 (1.0-1.8) | 5.3 (3.2-11.6) | | <0.001 |  |  |  |
|  |  | |  | |  |  | |  |  |  |  |
| Hepcidin (nmol/L) | 11.5 (5.8-25.2) | | 15.3 (6.6-26.4) | | 12.4 (6.3-27.0) | 9.3 (5.1-21.9) | | 0.034 |  |  |  |
|  |  | |  | |  |  | |  |  |  |  |
| Hepcidin-to-ferritin ratio | 53 (32-82) | | 53 (35-74) | | 49 (31-80) | 58 (35-91) | | <0.001 |  |  |  |
|  |  | |  | |  |  | |  |  |  |  |
| Ferritin (µg/L) | 241 (116-410) | | 22(12-408) | | 237 (98-472) | 269 (135-395) | | 0.61 |  |  |  |
|  |  | |  | |  |  | |  |  |  |  |
| Transferrin saturation (%) | 23 (17-30) | | 26 (20-33) | | 22 (18-31) | 19 (15-26) | | <0.001 |  |  |  |
|  |  | |  | |  |  | |  |  |  |  |
| ESA therapy, n (%) | 265 (70) | | 70 (56) | | 95 (76) | 99 (77) | | <0.001 |  |  |  |
|  |  | |  | |  |  | |  |  |  |  |
| Weekly ESA dose (IU/kg/w) | 75 (49-115) | | 56 (37-88) | | 64 (49-96) | 96 (61-145) | | <0.001 |  |  |  |
| Data are presented as median (interquartile range), or as absolute numbers (%).  ^a^Based on clinical history or presence of cardiovascular disease at enrollment.  ^b^Estimated glomerular filtration rate based on the chronic kidney disease epidemiology collaboration(CKD-EPI) equation for patients without dialysis and on the mean of renal creatinine and urea clearance from 24-hour urine collection in patients with peritoneal dialysis. PEW_SGA,_ protein energy wasting as defined by subjective global assessment (SGA); ESA, erythropoiesis stimulating agents; RAS inhibitors; renin angiotensin system inhibitors. | | | | | | | | |  |  |  |
|  |  |  |  |  |  |  |  |  |  |  |  |
|  |  |  |  |  |  |  |  |  |  |  |  |
|  |  |  |  |  |  |  |  |  |  |  |  |
|  |  |  |  |  |  |  |  |  |  |  |  |

| **Table S2. Levels of S-erythroferrone according to various categorical factors** | | | | |
| --- | --- | --- | --- | --- |
| **Categorical factor** |  | **n** | **Erythroferrone (ng/mL),**  **median (IQR)** | **p-value** |
| Gender | Women | 118 | 1.4 (0.5 – 3.4) | 0.94 |
|  | Men | 259 | 1.5 (0.5 – 3.3) |  |
| PD | Yes | 75 | 2.0 (0.8-5.0) | **0.01** |
|  | No | 302 | 1.3 (0.4-3.2) |  |
| ESA therapy | Yes | 265 | 1.7 (0.6 – 3.8) | **<0.01** |
|  | No | 112 | 0.7 (0.3 – 2.8) |  |
| High EHRI (> 1.07 IE/kg/w/g/L) | Yes | 127 | 1.07 (0.87 – 1.50) | **0.01** |
|  | No | 127 | 0.44 (0.34 – 0.53) |  |
| Iron therapy | Yes | 154 | 1.8 (0.8 – 5.1) | **<0.01** |
|  | No | 222 | 1.1 (0.4 – 3.0) |  |
| CVD | Yes | 127 | 2.0 (0.7 – 5.1) | **<0.01** |
|  | No | 246 | 1.2 (0.4 – 2.9) |  |
| DM | Yes | 112 | 1.5 (0.6 – 5.1) | 0.10 |
|  | No | 261 | 1.4 (0.5 – 3.0) |  |
| PEW | Yes | 94 | 2.3 (0.8 – 5.6) | **<0.01** |
|  | No | 261 | 1.2 (0.4 – 3.2) |  |
| PD: peritoneal dialysis; ESA: erythropoiesis stimulating agents; EHRI: ESA hyporesponsiveness index defined as the weekly ESA dose per kilogram of body weight divided by the hemoglobin level (g/L); CVD: cardiovascular disease; DM: diabetes mellitus; PEW: protein energy wasting as defined by subjective global assessment (SGA) >1. | | | | |

| **Table S3**. **Univariate Spearman’s rank correlations between erythroferrone and markers of relevance at baseline** | | | | | | | | | | | | | | | | | | | |
| --- | --- | --- | --- | --- | --- | --- | --- | --- | --- | --- | --- | --- | --- | --- | --- | --- | --- | --- | --- |
|  | Age | Hb | TIBC | RET-C | FER | TSAT | Iron | HEP | HEP/  FER | ESA | ALB | CRP | IL-6 | GFR | fT3^a^ | fT4^a^ | fT3/  fT4^a^ | HGS% | BMI |
| ERFE,  ng/mL | **0.20** | -0.09 | - 0.03 | **0.20** | 0.02 | **-0.24** | **-0.27** | **-0.17** | **-0.32** | **0.31** | **-0.17** | **0.24** | **0.23** | **-0.18** | 0.02 | 0.14 | **-0.20** | **-0.20** | 0.00 |
| Age,  years | - | **0.11** | **0.11** | 0.00 | 0.01 | 0.00 | 0.02 | -0.01 | -0.02 | -0.06 | **-**0.10 | **0.23** | **0.21** | **-**0.02 | **0.14** | **0.27** | **-0.33** | **-0.42** | **0.15** |
| Hb,  g/L |  | **-** | **0.30** | **0.12** | **-0.16** | **0.25** | **0.37** | **-0.16** | 0.04 | **-0.17** | **0.31** | **-0.22** | **-0.24** | **0.44** | **0.38** | **0.18** | -0.07 | **0.15** | **0.11** |
| TIBC,  µ/L |  |  | **-** | 0.01 | **-0.33** | **-0.19** | **0.23** | **-0.35** | -0.08 | **-0.24** | **0.41** | **-0.20** | **-0.27** | **0.26** | **0.34** | **0.36** | **-0.28** | **0.12** | **0.12** |
| RET-C,  10^9^/L |  |  |  | **-** | -0.01 | -0.04 | -0.04 | **-0.23** | **-0.32** | **0.22** | 0.10 | **0.11** | 0.08 | **0.15** | **-0.35** | **-0.27** | **0.20** | 0.05 | 0.10 |
| FER,  µ/L |  |  |  |  | **-** | **0.21** | 0.06 | **0.75** | **-0.17** | **-**0.02 | 0.01 | **0.19** | **0.17** | **-0.28** | 0.00 | 0.03 | 0.01 | -0.06 | 0.04 |
| TSAT,  % |  |  |  |  |  | **-** | **0.90** | **0.18** | 0.05 | **-0.21** | **0.13** | **-0.31** | **-0.33** | **0.17** | **0.19** | -0.08 | **0.19** | **0.12** | -0.08 |
| Iron,  µmol/L |  |  |  |  |  |  | **-** | 0.04 | 0.04 | **-0.31** | **0.33** | **-0.42** | **-0.45** | **0.30** | **0.33** | 0.06 | 0.09 | **0.20** | -0.01 |
| HEP,  nmol/L |  |  |  |  |  |  |  | - | **0.48** | -0.11 | -0.08 | **0.26** | **0.24** | **-0.31** | -0.03 | 0.00 | **-**0.03 | -0.05 | 0.04 |
| HEP /  FER |  |  |  |  |  |  |  |  | - | **-0.21** | **-**0.08 | **0.13** | 0.08 | -0.06 | -0.03 | -0.08 | 0.01 | 0.05 | 0.03 |
| ESA, IU/kg//w |  |  |  |  |  |  |  |  |  | **-** | -**0.13** | **0.21** | **0.22** | -0.11 | -0.10 | 0.03 | -0.10 | -0.10 | -**0.20** |
| ALB,  g/L |  |  |  |  |  |  |  |  |  |  | **-** | **-0.30** | **-0.44** | **0.36** | 0.06 | 0.06 | 0.12 | **0.30** | 0.05 |
| CRP,  mg/L |  |  |  |  |  |  |  |  |  |  |  | - | **0.58** | **-0.17** | **-0.19** | 0.02 | **-0.14** | **-0.20** | 0.09 |
| IL-6, pg/mL |  |  |  |  |  |  |  |  |  |  |  |  | - | **-0.36** | **-0.20** | -0.08 | -0.09 | **-0.36** | 0.06 |
| GFR,  mL/min/1,73^2^ |  |  |  |  |  |  |  |  |  |  |  |  |  | - | - | **-** | - | **0.29** | 0.08 |
| fT3^a^ |  |  |  |  |  |  |  |  |  |  |  |  |  |  | - | **0.67** | **-0.42** | -0.03 | 0.13 |
| fT4^a^ |  |  |  |  |  |  |  |  |  |  |  |  |  |  |  | - | **-0.89** | -0.05 | 0.02 |
| fT3/fT4^a^ |  |  |  |  |  |  |  |  |  |  |  |  |  |  |  |  | - | 0.09 | -0.02 |
| HGS %, % |  |  |  |  |  |  |  |  |  |  |  |  |  |  |  |  |  | - | 0.06 |
| Bold denotes p<0.05. **^a^**The Spearman's rank correlation coefficient was not calculated for the association between GFR and thyroid status since the assessment of the latter was not performed in patients with CKD 3-4 (GFR (≥15 mL/min/1.73m^2^). Hb, B-hemoglobin; TIBC, Total iron binding capacity; RET-C, reticulocyte count; FER, S-ferritin; TSAT, transferrin saturation; HEP, S-hepcidin; HEP/FER, hepcidin-to-ferritin ratio; ESA, the weekly dose of erythropoiesis stimulating agents; ALB, P-albumin; CRP, C-reactive protein; IL-6, S-interleukin-6; GFR, glomerular filtration rate as defined by either high GFR (≥15 mL/min/1,73m^2^) or low GFR (<15 mL/min/1.73m^2^); fT3, free triiodothyronine; fT4, free thyroxine; fT3/fT4 ratio; the free triiodothyronine / free thyroxine ratio; HGS%, handgrip strength %; BMI, body mass index. | | | | | | | | | | | | | | | | | | | |

| **Table S4. Multiple linear regression with log-normalized S-erythroferrone as dependent variable in 148 CKD patients with available measurements of the fT3/fT4 ratio**  **95 % Confidence interval** | | | | |  |
| --- | --- | --- | --- | --- | --- |
| **Variable** | **β** | **Lower limit** | **Upper limit** | **p-value** | **VIF** |
| log age | 0.16 | -0.02 | 1.56 | 0.055 | 1.2 |
| Gender | -0.01 | -0.20 | 0.17 | 0.854 | 1.0 |
| PD, yes/no | 0.07 | -0.12 | 0.29 | 0.425 | 1.5 |
| log RET-C | 0.23 | 0.23 | 1.32 | **0.006** | 1.3 |
| log Hepcidin-to-ferritin ratio | -0.24 | -0.77 | -0.14 | **0.005** | 1.3 |
| log CRP | 0.09 | -0.08 | 0.26 | 0.301 | 1.3 |
| log TSAT | -0.14 | -0.83 | 0.05 | 0.078 | 1.2 |
| log T3/T4 ratio | -0.20 | -0.59 | -0.040 | **0.025** | 1.5 |
| Adjusted R^2^ = 0.23; n =148.  Regression analyses with log-normalized S-erythroferrone as dependent variable.  β: standardized coefficient beta; PD: peritoneal dialysis; RET-C: reticulocyte count; CRP: high-sensitivity C-reactive protein; TSAT: transferrin saturation; fT3/fT4: the ratio of free triiodothyronine to free thyroxine; VIF: Variance Inflation Factor. | | | | |  |

**Table S5a. Risk of all-cause mortality in CKD patients with high tertile of erythroferrone as compared to middle and low tertiles of erythroferrone**

|  | **s-HR** | **95 % CI** | **p-value** | **n** |
| --- | --- | --- | --- | --- |
| Model 1: crude analysis | 2.14 | 1.45-3.14 | 0.000 | 376 |
| Model 2: adjustment for CKD cohort | 1.88 | 1.28-2.76 | 0.001 | 376 |
| Model 3: additional adjustment for Framingham Risk Score | 1.64 | 1.10 – 2.46 | 0.016 | 375 |
| Model 4: additional adjustment for the presence of protein energy wasting as per SGA | 1.65 | 1.10 – 2.48 | 0.016 | 353 |
| Model 5: additional adjustment for transferrin saturation | 1.55 | 1.01-2.38 | 0.045 | 321 |
| Model 6: additional adjustment for C-reactive protein | 1.52 | 0.99 –2.34 | 0.055 | 321 |
| Subdistribution hazard ratio (s-HR) and 95 % confidence intervals when adjusting for various covariates. s-HR: subdistribution hazard ratio; SGA: subjective global assessment.  Additional adjustment for the weekly ESA-dose in the sub-group of patients treated with erythropoiesis-stimulating agents did not significantly influence the results: s-HR = 1.65 (95 % CI = 0.98 – 2.78): p = 0.060. | | | | |

**Table S5b. Risk of cardiovascular mortality in CKD patients with high tertile of erythroferrone as compared to middle and low tertiles of erythroferrone.**

|  | **s-HR** | **95 % CI** | **p-value** | **n** |
| --- | --- | --- | --- | --- |
| Model 1: crude analysis | 2.84 | 1.61-5.03 | 0.000 | 376 |
| Model 2: adjustment for CKD cohort | 2.42 | 1.37-4.28 | 0.002 | 376 |
| Model 3: additional adjustment for Framingham Risk Score | 2.29 | 1.26 – 4.17 | 0.007 | 375 |
| Model 4: additional adjustment for the presence of protein energy wasting as per SGA | 2.23 | 1.22 – 4.05 | 0.009 | 353 |
| Model 5: additional adjustment for transferrin saturation | 1.98 | 1.06-3.68 | 0.031 | 321 |
| Model 6: additional adjustment for C-reactive protein | 2.00 | 1.08 –3.71 | 0.028 | 321 |
| Subdistribution hazard ratio (s-HR) and 95 % confidence intervals when adjusting for various covariates. s-HR: subdistribution hazard ratio; SGA: subjective global assessment. Additional adjustment for the weekly ESA-dose in the sub-group of patients treated with erythropoiesis-stimulating agents modified the association between S-erythroferrone and cardiovascular mortality: s-HR = 1.92 (95 % CI = 0.97 – 3.84): p = 0.063. | | | | |

**Figure S1.** A modified Poisson regression was used to calculate the cubic spline curve and 95% confidence level for erythroferrone and its association with mortality. The cubic spline curve was adjusted for age and sex. HRs: hazard ratios.

**References of the supplementary material**

1. Ghanavatian S, Diep LM, Barany P, Heimburger O, Seeberger A, Stenvinkel P, Rohani M, Agewall S: **Subclinical atherosclerosis, endothelial function, and serum inflammatory markers in chronic kidney disease stages 3 to 4**. *Angiology* 2014, **65**(5):443-449.

2. Stenvinkel P, Heimburger O, Paultre F, Diczfalusy U, Wang T, Berglund L, Jogestrand T: **Strong association between malnutrition, inflammation, and atherosclerosis in chronic renal failure**. *Kidney Int* 1999, **55**(5):1899-1911.

3. Xu H, Cabezas-Rodriguez I, Qureshi AR, Heimburger O, Barany P, Snaedal S, Anderstam B, Helin AC, Carrero JJ, Stenvinkel P *et al*: **Increased Levels of Modified Advanced Oxidation Protein Products Are Associated with Central and Peripheral Blood Pressure in Peritoneal Dialysis Patients**. *Perit Dial Int* 2015, **35**(4):460-470.

4. Ganz T, Jung G, Naeim A, Ginzburg Y, Pakbaz Z, Walter PB, Kautz L, Nemeth E: **Immunoassay for human serum erythroferrone**. *Blood* 2017, **130**(10):1243-1246.

5. Kong SH, Kim JH, Park YJ, Lee JH, Hong AR, Shin CS, Cho NH: **Low free T3 to free T4 ratio was associated with low muscle mass and impaired physical performance in community-dwelling aged population**. *Osteoporos Int* 2020, **31**(3):525-531.

6. Pasqualetti G, Calsolaro V, Bernardini S, Linsalata G, Bigazzi R, Caraccio N, Monzani F: **Degree of Peripheral Thyroxin Deiodination, Frailty, and Long-Term Survival in Hospitalized Older Patients**. *J Clin Endocrinol Metab* 2018, **103**(5):1867-1876.

7. Wang K, Zhang D, Cao G, Wang C, Wang L, Zhao R, He Q, Hou X, Gong L, Chen L: **A Low Free T3 to Free T4 Ratio Is Associated with Sarcopenia in Euthyroid Patients with Type 2 Diabetes Mellitus**. *J Diabetes Res* 2022, **2022**:2305156.
