# Supplementary figures and images for: Erythroferrone is associated with the hepcidin-to-ferritin ratio and cardiovascular mortality in chronic kidney disease
Source: Clin Kidney J. 2026 Mar 9;19(5):sfag075. doi: 10.1093/ckj/sfag075 (PMC13136888; doi:10.1093/ckj/sfag075)

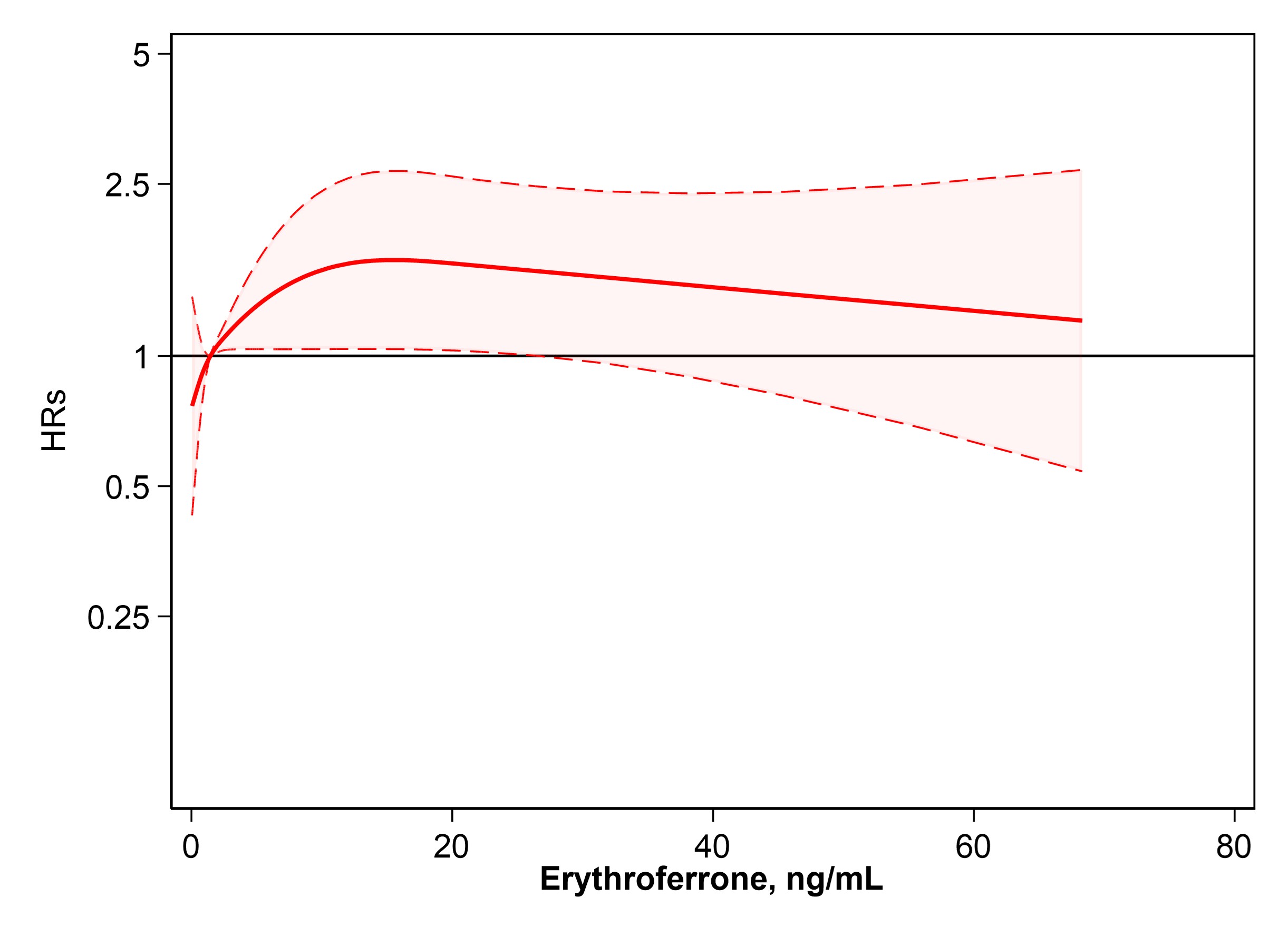

Supplement: sfag075_Supplemental_Files [file sfag075_supplemental_files.zip › Figure S1.jpg]
